# Enhancing U-Net for image denoising with bilateral filter noise residue and gradient estimation (BIRUNet)

**DOI:** 10.1038/s41598-025-30621-1

**Published:** 2025-12-07

**Authors:** S. Soniya, K. C. Sriharipriya, J. Christopher Clement, Umashankar Subramaniam

**Affiliations:** 1 School of Electronics Engineering, Vellore Institute of Technology, Vellore, India; 2 Prince Sultan University, Riyadh, Saudi Arabia

**Keywords:** Image denoising, Convolution neural network, Noise residue, Bilateral filter, Gradient information, Computational biology and bioinformatics, Engineering, Mathematics and computing

## Abstract

In recent years, Convolutional Neural Networks (CNNs) have achieved remarkable success in various computer vision tasks, including image denoising. Image denoising focuses on reconstructing a clean image from its noise-corrupted counterpart. In this paper, we propose BIRUNet, a bilateral-filter-based noise-residue U-Net enhanced with gradient estimation. The objective of this research is to improve the learning capability of the traditional U-Net by integrating manually derived image priors. Although several improved U-Net variants exist, many suffer from high computational cost and rely solely on learned noise patterns, which limits their reconstruction quality. To address these issues, BIRUNet incorporates two additional priors: (i) noise residue extracted using a traditional bilateral filter, and (ii) gradient information derived from the input image. These priors are concatenated with the noisy grayscale image and fed into an encoder-decoder U-Net architecture to generate a more accurate denoised output. The proposed model is evaluated both quantitatively and visually across multiple datasets. With a particular focus on preserving edge details, SSIM values are compared against those of more complex models, demonstrating superior performance. BIRUNet achieves a PSNR of 26.66 dB at a high noise level (σ = 50), confirming its effectiveness in challenging denoising scenarios.

## Introduction

Image denoising helps reduce compression artifacts, protect sensitive information, enhance visual quality, and improve the performance of subsequent image-processing tasks. Image restoration refers to techniques that minimize or eliminate degradations introduced during image acquisition. Noise and various forms of degradation can cause blurring, often arising from photometric or electronic disturbances. Blurring may also occur when the camera is out of focus or when bandwidth limitations distort the captured scene. Noise represents unwanted signals that diminish the visual quality of digital images.

Image denoising plays a crucial role in low-level vision for several reasons. First, noise is an unavoidable byproduct of the image-sensing process and can substantially degrade the visual quality of the captured image. Therefore, removing noise from observations is a fundamental step in many image-processing and computer-vision applications^1,2^. Second, image denoising serves as an ideal test bed for evaluating optimization strategies and image prior models from a Bayesian perspective^3,4^.

Discriminative learning approaches attempt to model image priors using pairs of noisy and ground-truth images. While some methods rely on brute-force learning strategies such as MLPs^5^ and CNNs^6,7^, others incorporate prior modeling within truncated inference frameworks^8^. Advances in CNN architecture design, training strategies, and representational capacity have led to notable improvements in denoising performance^9,10^. However, many existing learning-based models remain constrained by their optimization toward specific noise levels, limiting their generalization.

Furthermore, with the advent of deep learning, denoising performance has improved significantly. CNN-based approaches outperform traditional model-driven methods and offer faster inference, making them highly suitable for practical denoising applications.

**Fig. 1:**
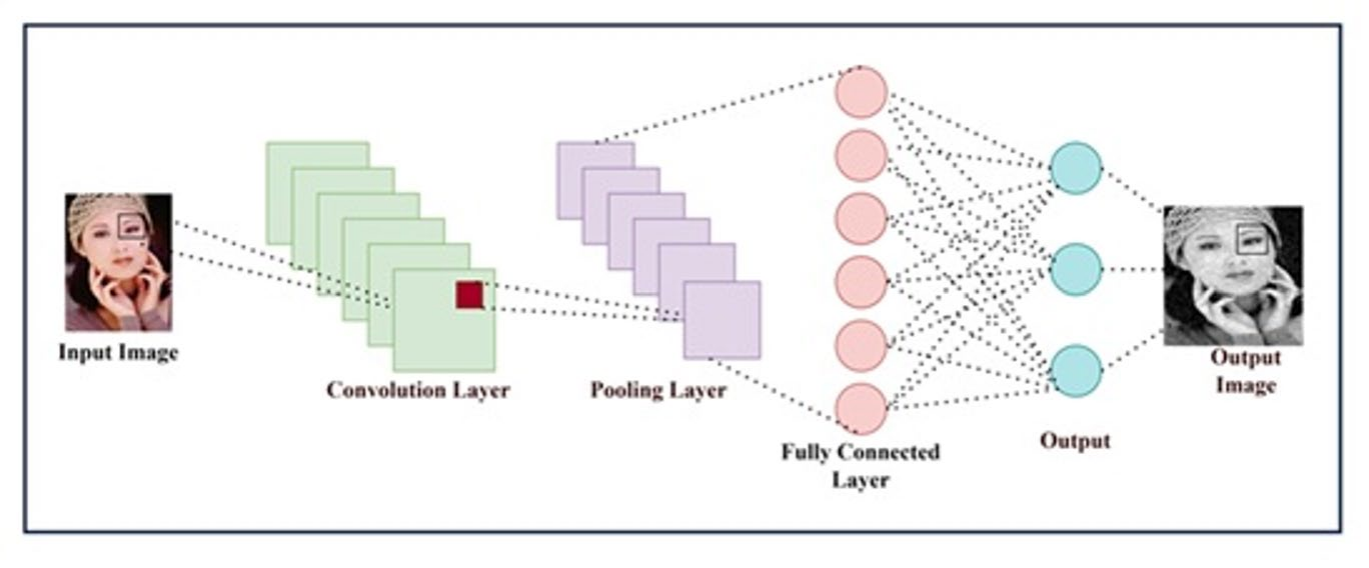
Fundamentals of CNN structure.

The basic structure of a CNN is shown in Fig. 1, illustrating how convolution operations are performed. As CNN architectures have evolved, the number of parameters has increased substantially, resulting in greater computational complexity. Traditional model-based denoising techniques such as Non-Local Means (NLM)^11^, Block-Matching and 3D Filtering (BM3D)^12^, and Weighted Nuclear Norm Minimization (WNNM)^13^ rely on explicit image prior modelling and often require time-consuming optimization procedures.

CNNs have been widely adopted for image denoising due to their strong performance in computer vision tasks^14,15^. The original U-Net architecture, introduced in^16^, is a fully convolutional network designed specifically for biomedical image segmentation, including applications such as liver and brain segmentation. U-Net consists of two main components: a contracting path (encoder) composed of convolutional layers and an expanding path (decoder) composed of up-convolution layers. Skip connections link corresponding encoder and decoder layers, enabling the concatenation of feature maps from the contracting path with those produced during up-sampling in the expanding path.

U-Net has been employed extensively in biomedical research. For instance, it has been used for cell segmentation^16^ and brain tumor detection and segmentation^17^. The 3D U-Net proposed in^18^ further extends the architecture to dense volumetric segmentation. Beyond segmentation, the U-Net can be adapted for various tasks by modifying its architecture. For example, in^19^, the U-Net was used as a generator for image-to-image translation tasks such as aerial-to-map conversion and grayscale-to-color transformation using adversarial learning. In^20^, the U-Net was applied for singing voice separation, where the magnitude of the audio spectrogram served as the input. Additionally, a Residual U-Net with incorporated residual blocks was used in^21^ to extract roads from aerial imagery.

A thorough analysis of current deep learning-based image denoising methods is presented in^22^, with a focus on the transition from conventional model-driven methods to data-driven architectures. In addition to analysing new developments like attention mechanisms, transformer-based architectures, and the difficulties of real-world noise modelling, their assessment emphasises the effectiveness, flexibility, and improved reconstruction capabilities of contemporary deep networks. The author of^23^ presented a multi-scale denoising architecture that uses curvelet thresholding specifically for noisy images found in the real world. Their method exhibits robust noise suppression while maintaining structural details and successfully captures multi-resolution geometric characteristics. Additionally, the study examines the wider applicability of curvelet-based approaches and contrasts their effectiveness with other traditional and learning-based methods, demonstrating better outcomes under challenging noise conditions.

Thorough reviews of contemporary research (2018–2023) have emphasised the variety of deep learning methods, such as blind denoising, hybrid approaches, optimization-based strategies for actual noise, and discriminative models for Gaussian noise. These studies highlight the advantages, drawbacks, and difficulties of current techniques, with a focus on over-smoothing, edge erosion, and artefacts at higher noise levels^24^. These studies collectively drive the development of hybrid models, like the suggested BIRUNet, which combines U-Net architectures with handcrafted priors (bilateral filter residue and gradient information) to improve overall denoising performance, edge preservation, and structural fidelity.

The main contribution of the proposed work is,

(i) The integration of two manually derived prior - bilateral filter noise residue and gradient information to enrich the U-Net input and overcome the limitations of relying solely on learned noise features.
(ii) The ability to enhance denoising performance without increasing model complexity, offering a lightweight alternative to deeper or more computationally intensive U-Net variants.
(iii) Improved preservation of structural and edge information, demonstrated through superior SSIM and PSNR performance at high noise levels.

## Related works

Traditionally, the two main techniques for image denoising were filtering and wavelet transforms. A new method for denoising digital images is machine learning, which has emerged recently.

Nowadays, image-denoising algorithms have improved in performance due to convolutional neural networks (CNNs) growing popularity. Prominent neural networks for noise reduction are DnCNN^6^, and IRCNN^7^. As ground truth noise, relative to the original clean image, is fed into the loss function, DnCNN, and Instead of the denoised image, IrCNN predicts the residue that is present in the image. Despite using batch normalization, ReLU activations, and repeated convolutional blocks in their basic architecture, both networks produced better results. Moreover, IrCNN^7^ and DnCNN^6^ rely on noise that is predicted blindly that is without considering the underlying structures and textures of the noisy image.

To minimize computation and prevent overfitting, CNNs typically employ pooling layers. This indicates that as information travels forward, feature map sizes get smaller. Initially, CNNs were primarily used to solve classification problems by providing a single label for each category. But unlike image processing tasks (like image segmentation and denoising), which aim for outputs that are almost the same size as the inputs, classification problems are very different^25^. In order to eliminate a broad range of noise levels and a spatially variable image, the FFDNet in^9^ was presented. This fast and flexible denoising network uses the noisy image and the noise level map as inputs. The input image is subjected to the down-sampling operator to maximize network processing speed. An additional input, the noise level map, is provided to manage the trade-off between maintaining image information and noise reduction. To eliminate Gaussian noise from images, the BRDNet network was proposed in^26^ and is made up of two subnetworks. To solve small mini-batch issues, they employed batch renormalization.

In^27^, the author employed the deformable convolution operation for image denoising to address the issue of using standard convolution, resulting in a changing training data distribution. Additionally, they increased the size of the receptive field by using dilated convolution. A dual network is suggested by DudeNet^28^ to extract complementary information and improve denoisers’ generalization capabilities. A feature attention mechanism is incorporated into RIDNet^29^ to select key features. These methods attempt to achieve denoising effects of higher quality but are limited by the well-known shortcomings in per-pixel loss functions.

Traditional and deep learning-based approaches to image denoising have been thoroughly studied, with more recent developments concentrating on enhancing computing efficiency and structural preservation. Although early deep learning techniques like DnCNN, FFDNet, and MLP-based architectures showed great promise in learning intricate noise patterns, they frequently lacked resilience in high noise environments. For example, in^30^, Annavarapu and Borra (2024) suggested a deep convolutional network-based figure-ground segmentation-based medical image denoising model to improve contextual feature separation between background noise and regions of interest. Similarly, in^31^, Annavarapu and Borra (2024) presented a CNN denoising framework based on adaptive watershed segmentation, where region-based priors enhanced the model’s capacity to recover fine structural features and edges. In addition, Annavarapu et al. (2023) created a hybrid BM3D collaborative filtering method that preserved the integrity of medical images while achieving noise reduction by combining block-matching and deep learning^32^.

By considering the balance of complexity and performance, this paper suggests an image-denoising technique based on U-Net with the bilateral filter to address the issues mentioned earlier. According to experimental verification, this technique effectively reduces image noise, improves image accuracy, clarifies image details, and lessens the challenge of further image processing.

## Materials and methods

Recently, many CNN architectures have been developed for Image Denoising. In general, the denoiser performance is directly correlated with the quality of the denoised image. This research aims to design a simpler network with a higher-quality image. In this work, we propose an enhanced version of the U-Net model for image denoising, named the Noise Residue from Bilateral Filter with Gradient Estimation Network (BIRUNet), which achieves improved denoising performance while preserving edge and structural details.

### Network architecture

**Fig. 2:**
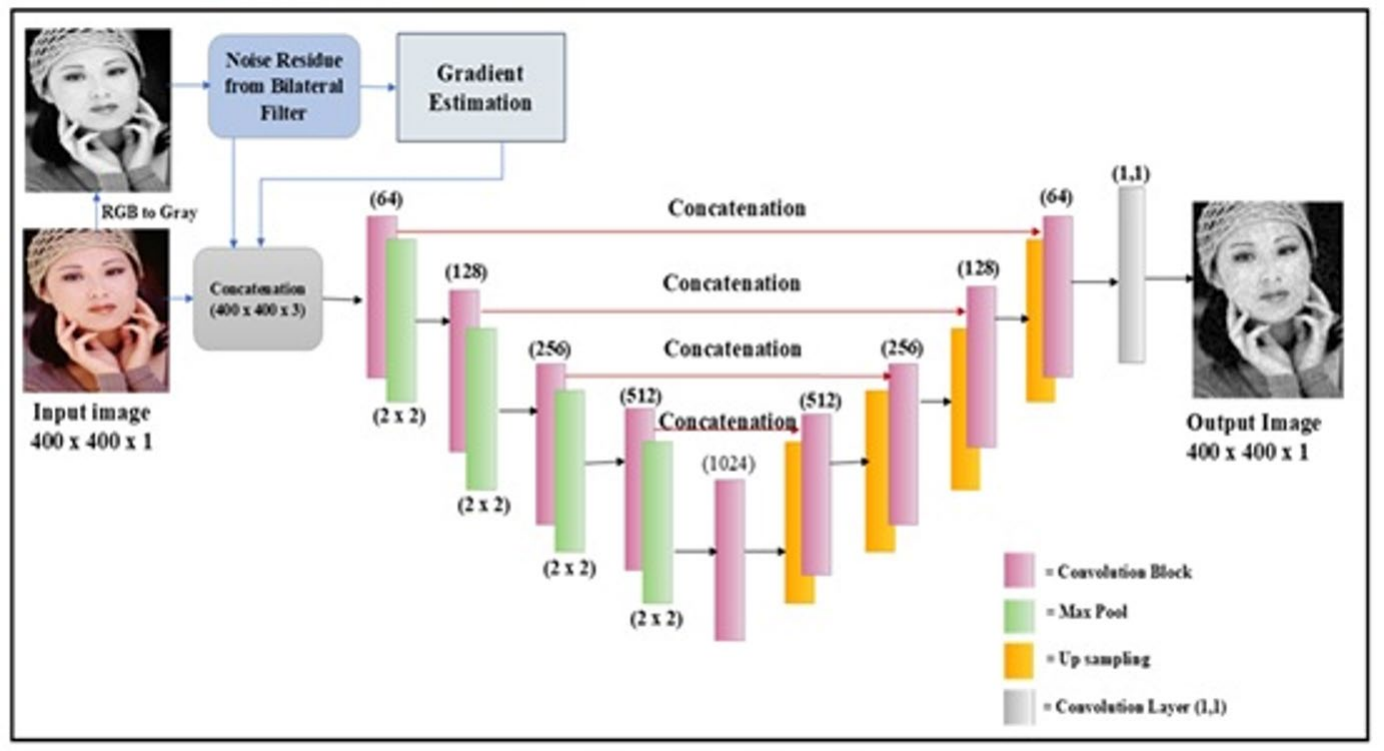
Structure of the proposed network design.

As shown in Fig. 2, the input image is resized to 400 × 400 pixels and converted from RGB to grayscale. The objective of this work is to enhance the learning capability of the conventional U-Net model by incorporating manually derived features.

Even though U-Net models have been improved, their main drawback is the high computational cost. Moreover, learning the noise pattern alone is not sufficient for reconstructing an improved denoised image, as other factors are also needed. By enhancing this, the noise pattern obtained from the conventional bilateral filter was applied in addition to the noisy gray image. Additionally, the gradient information from the image is applied to the model as well. As a result, the input gray image is concatenated with two evaluated image priors. Depending on this, the encoding and decoding of the U-Net structure is designed to produce the denoised image.

**Fig. 3:**
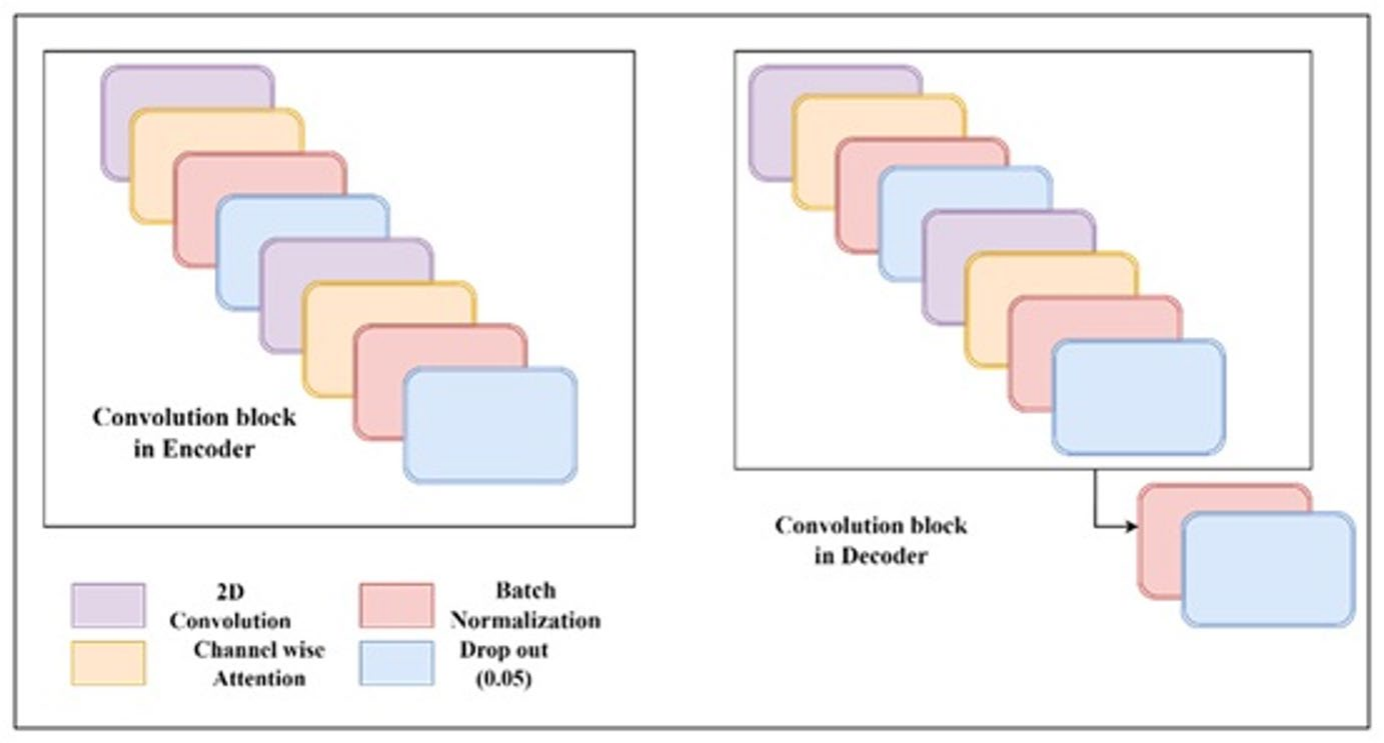
Convolution block layers in the encoder and decoder.

The Rectified Linear Unit (ReLU) activation function is used in every convolutional layer. However, the “sigmoid” function was used to activate the final convolution layers, producing the final denoised image. To enhance the denoising performance, dropout layers were also incorporated into the convolutional layers of the decoder and encoder. For the Decoder and Encoder stages, a dropout of 0.05 was used. Figure 3 illustrates how the convolution blocks are used to construct the encoder and decoder sections. Internally, there are two convolutional layers in each convolution block. Each encoder block has 64, 128, 256, and 512 filters for blocks 1, 2, 3, and 4 in the downstream, respectively.

The encoders are capable of convolution and downsampling, which are carried out by pooling layers and kernel filters, respectively. Unlike encoders, decoders are built by concatenating the features from the encoder stage and using up-samplers. Instead of a class label, the final result is a denoised image, in contrast to the classification models.

Let $\:B\left(x,\:y\right)$ be the clean image and $\:Bn\:\left(x,\:y\right)$ be the denoised image represented in Eq. (1)

1 $$\:Bn\:\left(x,\:y\right)=\:B\:\left(x,\:y\right)+\:Noise\left(0,\:\sigma\:\right)$$

The following formulation can be used to process a noisy image Y using bilateral filtering:

2 $$\:{\widehat{B}}_{x,y}=\sum\:_{P=x-R}^{x+R}\sum\:_{\text{q}=\text{y}-\text{R}}^{\text{y}+\text{R}}H\left(x,y;p,q\right){Y}_{p,q}\:\:\:\:\forall\:\left(p,q\right)\in\:{\varOmega\:}_{x,y}^{R}$$

Where, $\:{\widehat{B}}_{x,y}$ indicates the processed pixel of (x, y),

$\:H\left(x,y;p,q\right)$ denotes the weight coefficient relating to the neighboring pixel and the current pixel.

$\:{\:\:\:\varOmega\:}_{x,y}^{R}$ represents as a set of pixels in (2R + 1) x (2R + 1) centered window on (x, y).

The weight coefficient is represented as (3),

3 $$\begin{gathered} H~\left( {x,y;p,q} \right) = ~if\left( {p,q} \right)\Omega _{{x,y}}^{R} ~~~~~~~~w_{{x,y}}^{{ - 1}} \exp ~\left( { - \frac{{\left( {p - x} \right)^{2} + \left( {q - y} \right)^{2} }}{{2\sigma _{d}^{2} }}} \right)\exp \left( { - \frac{{\left( {y_{{p,q}} - y_{{x,y}} } \right)^{2} }}{{2\sigma _{r}^{2} }}} \right) \hfill \\ \,\,\,\,\,\,\,\,\,\,\,\,\,\,\,\,\,\,\,\,\,\,\,\,\,\,\,\,\,\,0\,\,\,\,\,\,\,\,\,\,\,\,\,\,\,\,\,\,\,\,\,\,\,\,\,\,\,\,\,\,\,\,\,\,\,\,\,\,\,\,\,\,\,\,\,\,\,\,\,\,\,\,\,\,\,Otherwise \hfill \\ \end{gathered}$$

Where, $\:{\sigma\:}_{d}$ is the domain standard deviation.

$\:{\sigma\:}_{r}$ is the range of Gaussian filters.

The normalization factor $\:{w}_{x,y}$is used to ensure that the filter maintains the image’s average gray value constant is given by (4)

4 $$\:{w}_{x,y}=\sum\:_{P=x-R}^{x+R}\sum\:_{q=y-R}^{y+R}\text{exp}\:\left(-\frac{{\left(p-x\right)}^{2}+{\left(q-y\right)}^{2}}{2{\sigma\:}_{d}^{2}}\right)exp\left(-\frac{{\left({y}_{p,q}-{y}_{x,y}\right)}^{2}}{{z}_{r}^{{\sigma\:}^{2}}}\right)$$

This work is inspired by^33^. Bilateral Filter represents a modified low-pass Gaussian filter for both the domain and range filters. The domain low-pass Gaussian filter gives large weights to pixels that are physically close to the center pixel. When using a range low-pass Gaussian filter, pixels that resemble the gray value’s center pixel are given large weights. Since the Bilateral Filter not only uses the fundamental gray filtering function but also describes the spatial arrangement of pixels, it is an effective way to remove Gaussian noise. Furthermore, the BF top reserve edge structures are partially enabled because of the range deviations $\:{\sigma\:}_{r}$ across an edge are comparatively larger than those along the edge. The Gaussian noise is filtered through a window of size 3 × 3. Equation (5) illustrates the approximate version of the noise matrix that is obtained.

5 $$\:Noise\:\left(\:x,\:y\right)=\:{B}_{noisy}\left(x,\:y\right)-\:{B}_{denoised}\left(x,\:y\right)$$

Let $\:{B}_{d}\:$(x, y) represents the image that was extracted using the gradient magnitude image $\:{G}_{m}$(x, y) and the Bilateral filter. The gradient magnitude function has been mentioned in Eq. (6)

6 $$\:{G}_{m}(x,\:y)=\:\sqrt{({{{G}_{m}}_{x}(\text{x},\:\text{y})}^{2}\:+\:{{{G}_{m}}_{y}(\text{x},\:\text{y})}^{2})}\:$$

Using Sobel operators, the following formulas are used to compute the vertical gradient $\:{{G}_{m}}_{y}$(x, y) (5b) and the horizontal gradient $\:{{G}_{m}}_{x}$ (x, y) (5a):

6a $$\begin{aligned} G_{{m_{x} }} ~\left( {x,~y} \right) & = ~\left( {B_{d} \left( {x + 1,~y - 1} \right) + ~2~B_{d} ~\left( {x + 1,~y} \right) + B_{d} \left( {x + 1,~y + 1} \right)} \right) \\ & - ~\left( {B_{d} ~\left( {x - 1,~y - 1} \right) + ~2B_{d} ~\left( {x - 1,~y} \right) + ~B~\left( {x - 1,~y + 1} \right)} \right) \\ \end{aligned}$$

6b $$\begin{aligned} G_{{m_{y} }} \left( {x,~y} \right) & = ~\left( {B_{d} ~\left( {x - 1,~y - 1} \right) + ~2~B_{d} ~\left( {x,~y - 1} \right) + ~B_{d} ~\left( {x + 1,~y - 1} \right)} \right) \\ & - ~\left( {B_{d} ~\left( {x - 1,y + 1} \right) + ~2~B_{d} ~\left( {x,~y + 1} \right) + B_{d} ~\left( {x + 1,~y + 1} \right)} \right) \\ \end{aligned}$$

Therefore, three layers of information are applied to the model, namely the input gray image, the predicted noisy image from Eq. (5), and finally the gradient information from Eq. (6). Following this, encoding and decoding of the U-Net are targeted to give a denoised image.

## Evaluation metrics

### Mean squared error (MSE)

The acronym for Mean Squared Error is MSE. The MSE can be defined as follows: it measures the squared difference between the actual target and the predicted image.

7 $$\:MSE\:loss=\:\frac{1}{M\:\times\:\:N}\:\sum\:_{i=1}^{M}\sum\:_{j=1}^{N}{\left({predicted}_{i,\:j}-{actual\:target}_{i,\:j}\:\right)}^{2}$$

### Peak signal to noise ratio (PSNR)

PSNR is referred to as Peak signal to Signal-to-Noise Ratio. It indicates the quality of an image. It is the ratio of the maximum value of the pixel to the noise. For higher values, lower the error and expressed in the logarithmic decibel scale.

8 $$PSNR = ~10\log \frac{{Max^{2} }}{{MSE~loss}}dB$$

### Structural similarity index (SSIM)

To calculate the similarity index between two images, utilize SSIM, which ranges between 0 and 1. It is used to quantify the image quality degradation.

9 $$\:SSIM\:\left(x,y\right)=\:\:\frac{\left(2{\mu\:}_{x}{\mu\:}_{y}+{c}_{1}\right)\left(2{\sigma\:}_{xy}+{c}_{2}\right)}{\left({\mu\:}_{x}^{2}+{\mu\:}_{y}^{2}+{c}_{1}\right)\left({\sigma\:}_{x}^{2}+{\sigma\:}_{y}^{2}+{c}_{2}\right)}\:\:\:\:\:\:\:\:\:\:\:\:\:\:\:\:\:\:\:\:\:\:\:\:\:\:\:\:\:\:\:\:\:\:\:\:\:\:\:\:\:$$

## Datasets

For training and testing purposes, we have taken a dataset from the Berkeley Segmentation Dataset (BSD300). It consists of 300 images split as 80% of training and 20% of testing, and validation, that is 240 images for training, and the remaining 30/30 images for testing and validation. After randomly arranging the image names into 42 states, the database was divided. The original scale for each image was changed from [0, 255] to within the range of [0, 1]. The actual dimensions of the images were 481 × 321 and 321 × 481 with the color channels of RGB, then resized to 400 × 400 in gray format with the nearest interpolation.

### Parameter settings

The model was first trained at a learning rate of 1 × 10⁻³, and based on validation performance utilising callback functions, it was adaptively lowered in non-linear steps of 1 × 10⁻⁵, 1 × 10⁻⁶, and 1 × 10⁻⁷. A 1 × 10⁻⁶ L2 regularisation was used to stabilise training and avoid overfitting. When there was still no improvement after ten patient attempts, this adaptive variation was carried out using the call-back functions. The Adam optimizer^34^ was employed to optimize the model. In the case of the BSD300 database, the batch size was modified to 4. For fifty epochs, this model was trained. Four images per batch were used in the training loop iterations to cover all training images. This work did not involve image augmentation because the primary goal is to enhance the traditional U-Net model’s learning capability.

### Experimental setup

Our model was trained on a NVIDIA GeForce GTX1650 graphical processing unit (GPU), and all experiments were implemented using Collab on a PC with Intel Core i5-10300 H, 8 GB RAM.

## Analysis of results

Using the PSNR and SSIM indices, we compare our model’s denoising performance to that of several sophisticated algorithms. Table 1 denotes the performance analysis of the BIRUNet architecture compared with the state-of-the-art methods. With noise levels of 50, 25, and 15, our proposed work yields a higher PSNR value of 26.66 dB, 29.56 dB, and 30.90 dB. A lower noise level of 15 gained a nearer PSNR value of 30.90 dB compared with benchmark models.

**Table 1:** Quantitative comparison of the BSD dataset with various models based on PSNR in dB.

Methods									
		BM3D^12^	DnCNN^6^	ADNet^35^	FFDNet^9^	IRCNN^7^	DudeNet^28^	LIGN^36^	BIRUNet
Noise levels	σ = 50	25.62	26.23	26.29	26.29	26.14	26.31	26.53	26.66
	σ = 25	28.57	29.23	29.25	29.19	29.25	29.29	29.42	29.56
	σ = 15	31.07	31.73	31.74	31.63	31.51	31.78	31.85	30.90

**Fig. 4:**
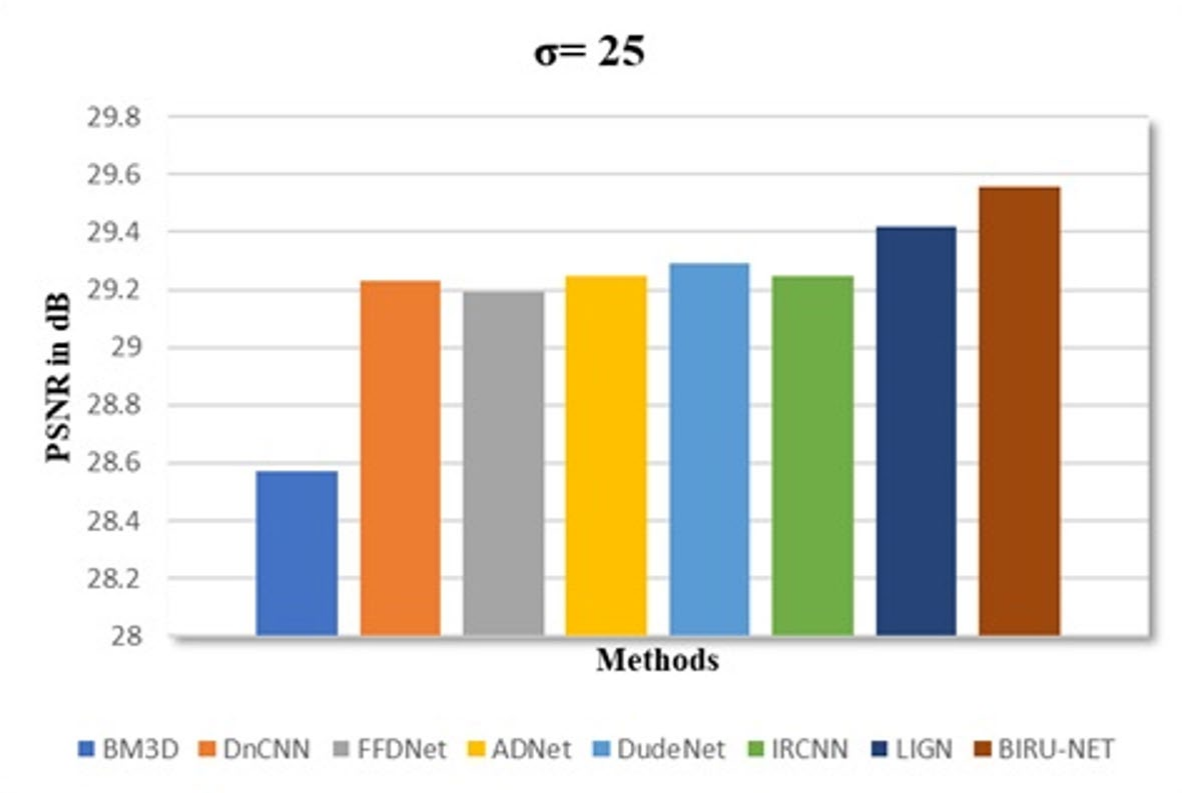
A visual depiction of the proposed work alongside a comparison with state-of-the-art techniques.

As referred from the comparison Table 1, we developed a graphical representation of the proposed work with complex architectures. Our BIRUNet architecture shows a higher value with a noise level of 25. Complex architectures of BM3D^12^, DnCNN^6^, FFDNet^9^, ADNet^35^, DudeNet^28^, IRCNN^7^, and LIGN^36^ are taken for the comparative analysis. A performance evaluation is shown in Fig. 4, where the proposed work demonstrated higher performance.

**Fig. 5:**
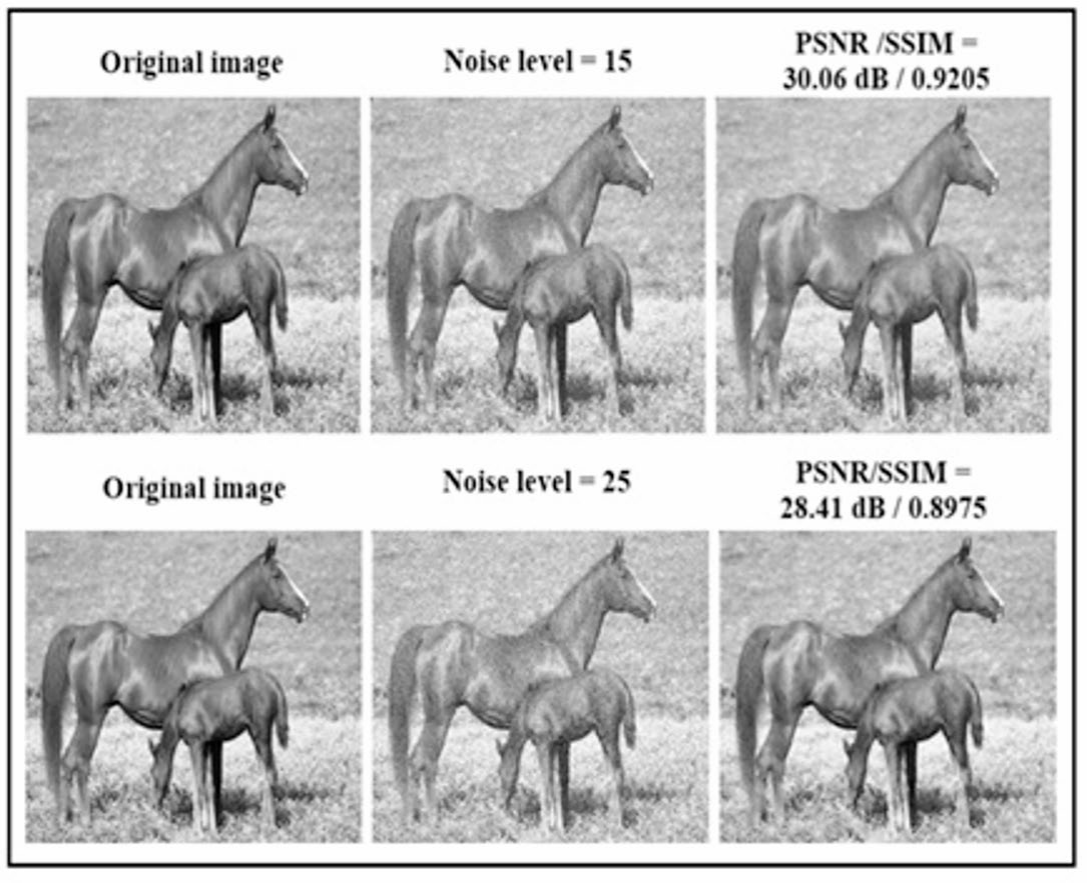
Results for Image denoising using BIRUNet architecture with noise levels of 15 and 25.

Restoring a noise-free image from a noise-corrupted image is a process of Image denoising. Denoising an image is not an easy task; a simple architecture of the Enhanced U-Net is used in the research. The learning capability of the network has been increased by the BIRUNet model results obtained in Fig. 5. Original image, Noisy image of 15 and 25, and finally denoised images are displayed.

As we mentioned earlier, using a bilateral filter not only removes noise from the images but also provides an edge-preserving and noise-reducing smoothing filter. Here, Table 2 shows the SSIM values that are Structural Similarity index values of BIRUNet, with the comparison of Conventional methods with the noise levels of 50, 25, and 15.

**Table 2:** Calculation of SSIM values with noise levels of 50, 25, and 15.

Models	σ = 50	σ = 25	σ = 15
EPLL^4^	0.6917	0.8125	0.8826
MLP^5^	0.7312	0.8432	0.8727
DnCNN^6^	0.7493	0.8802	0.9018
IrCNN^7^	0.7500	0.8562	0.9071
RIDNet^29^	0.7320	0.8890	0.9059
BIRUNet	0.7656	0.8913	0.9163

**Fig. 6:**
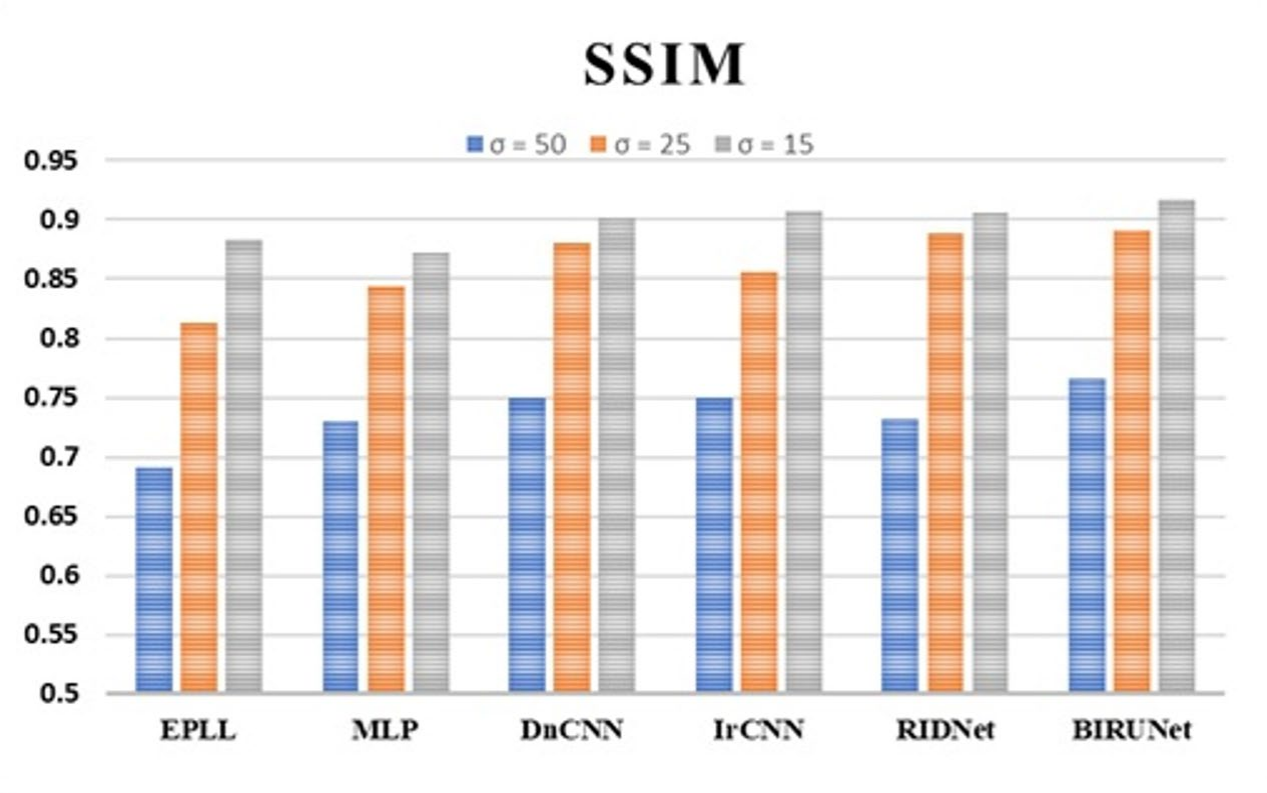
Graphical representation of SSIM values with three noise levels of 50, 25, and 15 respectively.

It displayed a performance analysis of SSIM value with the reference of EPLL^4^, MLP^5^, DnCNN^6^, IRCNN^7^, RIDNet^29^, and our proposed work, BIRUNet in Fig. 6. It provides information on image quality, a perceptual metric of image processing.

**Fig. 7:**
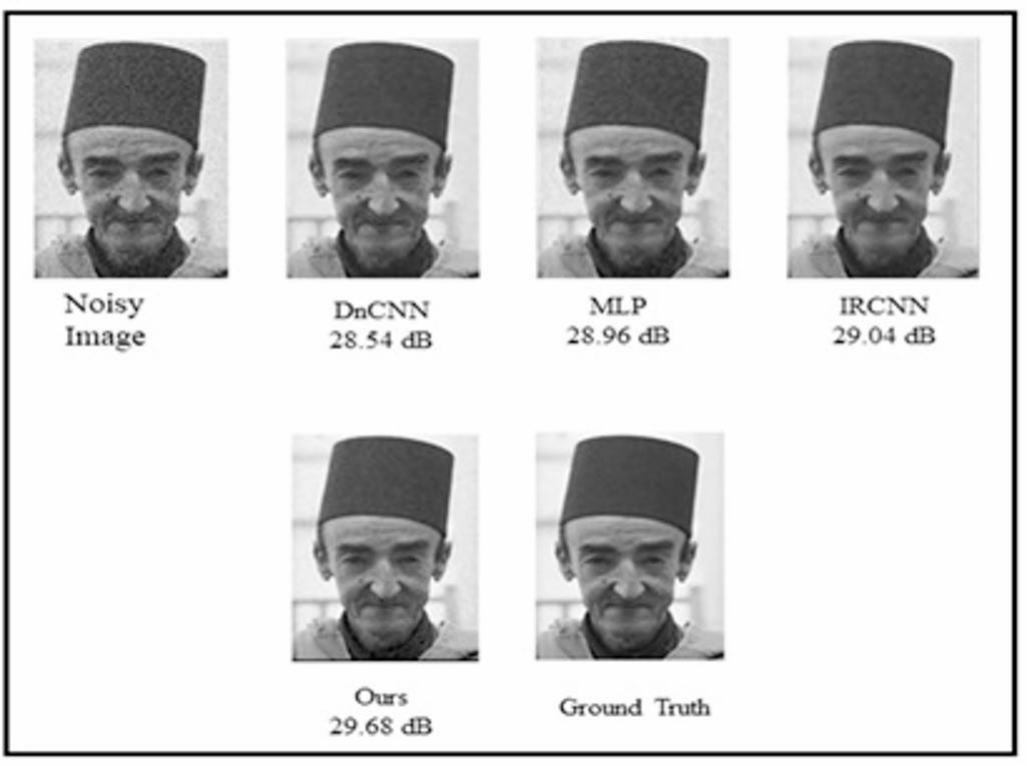
Results of denoising a chosen grayscale image from BSD at σ = 50 noise level.

From the BSD database, a grayscale image of 189080 to show a result of denoising using BIRUNet is displayed in Fig. 7. To analyze the denoising performance, DnCNN^6^, MLP^5^, and IRCNN^7^ models denoised the image is revealed and compared with the BIRUNet architecture. Figure 7 gained a PSNR value of 29.68 dB of our proposed work also provides a clean image.

**Table 3:** Comparison of our model’s denoising performance with a few benchmark models at different datasets such as CBSD68, KODAK24, and McMaster.

	KODAK24			McMaster			CBSD68		
σ	15	25	50	15	25	50	15	25	50
CBM3D^12^	34.28	31.68	28.46	34.06	31.66	28.51	33.50	30.69	27.37
DnCNN^6^	34.48	32.03	28.85	33.44	31.51	28.61	33.89	31.33	27.97
FFDNet^9^	34.63	32.13	28.98	34.66	32.35	29.18	33.87	31.21	27.96
ADNet^35^	34.76	32.26	29.10	34.93	32.56	29.36	33.99	31.31	28.04
GradNet^37^	34.85	32.35	29.23	34.81	32.45	29.39	34.07	31.39	28.12
BIRUNet	33.96	32.39	29.26	33.49	32.62	29.42	32.91	31.44	28.24

For testing purposes, we included different datasets available online they are CBSD68, KODAK24, and McMaster. Our proposed work performed well compared with the other models and was analyzed with the noise levels of 15, 25, and 50 in Table 3.

## Conclusion

The goal of this research is to increase the learning capacity of the conventional U-Net model by including manual features. Here, we used a limited dataset to train a network. There is no image augmentation was done. The noise pattern obtained from the conventional bilateral filter was applied in addition to the noisy gray image. Moreover, gradient information from the image is also applied to the model. Consequently, two assessed image priors are concatenated with the input grayscale image. This informs how the U-Net network is encoded and decoded to generate the denoised image. We present an enhanced U-Net of BIRUNet design that has been proven to have Greater performance in image denoising, for noise levels of 50 and 25, gaining a higher PSNR value compared to the state-of-the-art methods. We plan to work on real-time images and color images in the future.

## Data Availability

BSD dataset for training: [https://www2.eecs.berkeley.edu/Research/Projects/CS/vision/bsds/](https:/www2.eecs.berkeley.edu/Research/Projects/CS/vision/bsds)In order to Test, Datasets KODAK24, McMaster, CBSD68, and SET12 were used. The testing dataset was posted to Figshare after being downloaded from Kaggle. For convenience, a link to the dataset is included. [https://doi.org/10.6084/m9.figshare.26827765.v1](https:/doi.org/10.6084/m9.figshare.26827765.v1)This study uses only publicly available and fully anonymised dataset(s). No human subjects were recruited, and no identifiable personal information is included. Hence, informed consent was not required.

## References

[1] Andrews, H. C. and Bobby ray Hunt. *Digital Image Restoration*. (Prentice Hall Professional Technical Reference, 1977).

[2] Chatterjee, P. Is denoising dead? *IEEE Trans. Image Process.* **19** (4), 895–911 (2009). 19932997 10.1109/TIP.2009.2037087

[3] Roth, S. & Black, M. J. Fields of experts: A framework for learning image priors. In *IEEE Computer Society Conference on Computer Vision and Pattern Recognition (CVPR’05)*. **2** (IEEE, 2005).

[4] Zoran, D. & Yair Weiss. From learning models of natural image patches to whole image restoration. In *2011 International Conference on Computer Vision*. (IEEE, 2011).

[5] Burger, H. C., Christian, J. & Schuler and Stefan Harmeling. Image denoising: Can plain neural networks compete with BM3D? In *2012 IEEE Conference on Computer Vision and Pattern Recognition*. (IEEE, 2012).

[6] Zhang, K. et al. Beyond a Gaussian denoiser: residual learning of deep Cnn for image denoising. *IEEE Trans. Image Process.* **26**, 3142–3155 (2017). 28166495 10.1109/TIP.2017.2662206

[7] Zhang, K. et al. Learning deep CNN denoiser prior for image restoration. In *Proceedings of the IEEE Conference on Computer Vision and Pattern Recognition*. (2017).

[8] Chen, Y. & Pock, T. Trainable nonlinear reaction diffusion: A flexible framework for fast and effective image restoration. *IEEE Trans. Pattern Anal. Mach. Intell.* **39** (6), 1256–1272 (2016). 27529868 10.1109/TPAMI.2016.2596743

[9] Zhang, K., Zhang, L. & Wangmeng Zuo, and FFDNet: toward a fast and flexible solution for CNN-based image denoising. *IEEE Trans. Image Process.* **27** (9), 4608–4622 (2018). 10.1109/TIP.2018.2839891 29993717

[10] Guo, S. et al. Toward convolutional blind denoising of real photographs. In *Proceedings of the IEEE/CVF Conference on Computer Vision and Pattern Recognition*. (2019).

[11] Buades, A. & Coll, B. and J-M. Morel. A non-local algorithm for image denoising. In *IEEE Computer Society Conference on Computer Vision and Pattern Recognition (CVPR’05)*. **2**. (IEEE, 2005).

[12] Dabov, K. et al. Image denoising by sparse 3-D transform-domain collaborative filtering. *IEEE Trans. Image Process.* **16**, 2080–2095 (2007). 17688213 10.1109/tip.2007.901238

[13] Gu, S. et al. Weighted nuclear norm minimization with application to image denoising. In *Proceedings of the IEEE Conference on Computer Vision and Pattern Recognition*. (2014).

[14] Ghose, S., Singh, N. & Singh, P. Image denoising using deep learning: Convolutional neural network. In *2020 10th International Conference on Cloud Computing, Data Science & Engineering (Confluence)*. (IEEE, 2020).

[15] Li, X. et al. Detail retaining convolutional neural network for image denoising. *J. Vis. Commun. Image Represent.* **71**, 102774 (2020).

[16] Ronneberger, O., Fischer, P. & Brox, T. U-net: Convolutional networks for biomedical image segmentation. *Medical Image Computing and Computer-assisted Intervention–MICCAI: 18th international Conference, Munich, Germany, October 5–9, 2015, Proceedings, Part III 18*. (Springer International Publishing, 2015).

[17] Dong, H. et al. Automatic brain tumor detection and segmentation using U-Net based fully convolutional networks. In *Medical Image Understanding and Analysis: 21st Annual Conference, MIUA*. (Springer International Publishing, 2017).

[18] Çiçek, Ö. et al. 3D U-Net: learning dense volumetric segmentation from sparse annotation. In *Medical Image Computing and Computer-Assisted Intervention–MICCAI: 19th International Conference, Athens, Greece, October 17–21, 2016, Proceedings, Part II 19*. (Springer International Publishing, 2016).

[19] Isola, P. et al. Image-to-image translation with conditional adversarial networks. In *Proceedings of the IEEE Conference on Computer Vision and Pattern Recognition*. (2017).

[20] Jansson, A. et al. Singing voice separation with deep u-net convolutional networks. (2017).

[21] Zhang, Z., Liu, Q. & Wang, Y. Road extraction by deep residual u-net. *IEEE Geosci. Remote Sens. Lett.* **15** (5), 749–753 (2018).

[22] Jiang, B. et al. Eficient image denoising using deep learning: A brief survey. *Inf. Fus.* 103013. (2025).

[23] Panigrahi, S. et al. Multi-scale based approach for denoising real-world noisy image using curvelet thresholding: scope and beyond. *IEEE Access.* **12** : 25090–25105. (2024).

[24] Jebur, R. et al. A comprehensive review of image denoising in deep learning. *Multimedia Tools Appl.* **83** (20), 58181–58199 (2024).

[25] Wang, S. F. Multi-wavelet residual dense convolutional neural network for image denoising. *IEEE Access.* **8**, 214413–214424 (2020).

[26] Tian, C., Xu, Y. & Zuo, W. Image denoising using deep CNN with batch renormalization. *Neural Netw.* **121**, 461–473 (2020). 31629201 10.1016/j.neunet.2019.08.022

[27] Zhang, Q. et al. A robust deformed convolutional neural network (CNN) for image denoising. *CAAI Trans. Intell. Technol.* **8** (2), 331–342 (2023).

[28] Tian, C. et al. Designing and training of a dual CNN for image denoising. *Knowl. Based Syst.* **226**, 106949 (2021).

[29] Anwar, S. & Barnes, N. Real image denoising with feature attention. In *Proceedings of the IEEE/CVF International Conference on Computer Vision*. (2019).

[30] Annavarapu, A. Figure-ground segmentation based medical image denoising using deep convolutional neural networks. *Int. J. Comput. Appl.* **46** (12), 1179–1205 (2024).

[31] Annavarapu, A. & Borra, S. An adaptive watershed segmentation based medical image denoising using deep convolutional neural networks. *Biomed. Signal Process. Control*. **93**, 106119 (2024).

[32] Annavarapu, A. et al. A hybrid medical image denoising based on block matching 3D collaborative filtering. *SN Comput. Sci.* **5** (1), 35 (2023).

[33] Tomasi, C. & Manduchi, R. Bilateral filtering for gray and color images. In *Sixth international conference on computer vision (IEEE Cat. No. 98CH36271)*. (IEEE, 1998).

[34] Kingma, D. P. and Jimmy Ba. Adam: A method for stochastic optimization. https://arXiv.org/abs/1412.6980. (2014).

[35] Tian, C. et al. Attention-guided CNN for image denoising. *Neural Netw.* **124**, 117–129 (2020). 31991307 10.1016/j.neunet.2019.12.024

[36] Qiao, S. et al. Layered input GradiNet for image denoising. *Knowl. Based Syst.* **254**, 109587 (2022).

[37] Liu, Y. et al. Gradnet image denoising. In *Proceedings of the IEEE/CVF Conference on Computer Vision and Pattern Recognition Workshops*. (2020).

